# Differences in Anthocyanin Accumulation Profiles between Teinturier and Non-Teinturier Cultivars during Ripening

**DOI:** 10.3390/foods10051073

**Published:** 2021-05-12

**Authors:** Meng-Bo Tian, Lin Yuan, Ming-Yuan Zheng, Zhu-Mei Xi

**Affiliations:** 1 College of Enology, Northwest A&F University, Yangling 712100, Shaanxi, China; Tianmengbo@nwsuaf.edu.cn (M.-B.T.); starzmy@nwsuaf.edu.cn (M.-Y.Z.); 2 College of Food Science and Nutritional Engineering, China Agricultural University, Beijing 100083, China; yuanlin@cau.edu.cn; 3 Shaanxi Engineering Research Center for Viti-Viniculture, College of Enology, Northwest A&F University, Yangling 712100, Shaanxi, China

**Keywords:** grape, Yan 73, Dunkelfelder, anthocyanin, cluster analysis

## Abstract

Anthocyanins are vital components of plant secondary metabolites, and are also the most important coloring substances in wine. Teinturier cultivars are rich in anthocyanins. However, the differences in anthocyanin accumulation and profiles between teinturier and non-teinturier cultivars have not been reported. In this study, Yan 73 and Dunkelfelder were selected as the experimental materials, and three non-teinturier cultivars were used for comparison. LC-MS and qRT-PCR were used to determine the individual anthocyanin contents and the relative gene expression. The results show that the total anthocyanin content of the teinturier cultivars was considerably higher than that in non-teinturier cultivars, and the levels of individual anthocyanins increased gradually during ripening. Lower ratios of modified anthocyanins were found in the teinturier cultivars, which was not only due to the high expression level of *VvUFGT* and *VvGST4*, but also due to the relatively low expression of *VvOMT* in these cultivars. Cluster analysis of gene expression and anthocyanin accumulation showed that *VvUFGT* is related to anthocyanin accumulation, and that *AM1* is related to the synthesis and transport of methylated anthocyanins. Our results will be useful for further clarifying the pathways of anthocyanin synthesis, modification, and transport in teinturier cultivars.

## 1. Introduction

Anthocyanins are secondary plant metabolites that protect plants from UV radiation and low temperatures [1,2], and assist in response to drought stress and defense against microorganisms [3,4]. Most importantly, they impart various colors to plants as pigments.

The anthocyanin biosynthetic pathway of plants can be divided into two parts: the phenylpropanoid metabolic pathway and the flavonoid pathway. Several structural genes, such as *PAL*, *CHS*, *CHI*, *F3H*, *DFR*, *LDOX*, and *UFGT*, are involved in anthocyanin biosynthesis [5]. Among these, UFGT (UDP-glucose: flavonoid 3-*O*-glucosyltransferase) is the last enzyme for anthocyanin biosynthesis, which catalyzes anthocyanidin glycosylation to form anthocyanins [6]. Similar to other naturally occurring phenolic compounds, anthocyanins are mostly modified, and regulated by the products of several structural genes. OMT (*O*-methyltransferase) possibly plays a major role in anthocyanin methylation in grapes [7] because it can transfer a methyl group from *S*-adenosylmethionine (SAM) onto a hydroxyl group on anthocyanins [8]. The biosynthesis and modification of anthocyanins occur on the cytoplasmic face of the endoplasmic reticulum (ER) [9], following which, the anthocyanins are transported to vacuoles for storage and to perform their functions. Two major mechanisms of anthocyanin transport have been identified. One mechanism involves the transport mediated by glutathione S-transferases (GSTs). Several genes encoding GST have been identified in grapes, and *VvGST4* is believed to be involved in anthocyanin transport [10]. The expression level of *VvGST4* in grape skin during veraison correlates with anthocyanin accumulation, and the effect of *VvGST4* as a GST-anthocyanin transporter has been confirmed using complementation studies [10,11]. Another mechanism involves transportation by anthoMATE (AM), which is located at the tonoplast and mediates transport specifically of acylated anthocyanin, but not malvidin 3-*O*-glucoside or cyanidin 3-*O*-glucoside [12]. These two transport mechanisms may coexist in plants to constitute a complex anthocyanin transport system [13]. Finally, anthocyanins are transported into the vacuole and stored as anthocyanin vacuolar inclusions (AVIs) [14].

Grape anthocyanins have been the focus of research due to their critical effect on the quality of grapes and wines. The anthocyanin component and content in grape skins are variable and influenced by many factors; however, the genetic factors determine a specific anthocyanin pattern for each grape cultivar [15]. Zhao et al. [16] found 9910 mg/kg DW (dry weight) of anthocyanins in the skin of Cabernet Sauvignon and Samoticha et al. [17] found 5680 mg/kg DW in Pinot Noir. He et al. [18] found the anthocyanins of Syrah for two consecutive years to be 18,087 and 10,210 mg/kg DW, respectively. 

Teinturier cultivars not only accumulate anthocyanins in skins, but also in flesh. They also have much higher anthocyanin content in skins than non-teinturier cultivars, so have significant potential for wine blending. Anthocyanins in the flesh of teinturier cultivars have been reported [19]. The synthesis of anthocyanins occurred initially in the flesh and later in the skin, and the anthocyanin content of skin exceeded that in the flesh until 13 weeks after anthesis. However, the differences of anthocyanin accumulation and profiles between teinturier and non-teinturier cultivars have not been reported, and the expression levels of genes related to anthocyanin synthesis, modification, and transport have not been clarified yet.

In this study, two teinturier cultivars (‘Yan 73’ and ‘Dunkelfelder’) and three non-teinturier cultivars (‘Cabernet Sauvignon’, ‘Syrah’, and ‘Pinot Noir’) were selected. Harvest maturity parameters (RS, TA, and pH value) were evaluated, and the differences of anthocyanins profiles and gene expression levels between teinturier and non-teinturier cultivars were compared. The results will provide a theoretical reference for anthocyanin synthesis, modification, and transport of teinturier cultivars.

## 2. Materials and Methods

### 2.1. Chemicals

All of the chemical reagents used in the present study were analytical or HPLC grade. The Folin–Ciocalteu reagent was purchased from Solarbio (Beijing, China), p-DMACA was purchased from Aladdin (Shanghai, China), gallic acid and rutin were obtained from Sigma (St. Louis, MO, USA), acetonitrile and formic acid were obtained from Kemiou Chemical Reagent Co., Ltd. (Tianjin, China). Copper sulfate pentahydrate, concentrated hydrochloric acid (36–38%), sodium hydroxide, and glucose were supplied by Xilong Chemical Industry Co., Ltd. (Sichuan, China).

### 2.2. Plants and Fruit Samples

Teinturier cultivars Yan 73 and Dunkelfelder, and non-teinturier cultivars Cabernet Sauvignon, Pinot Noir, Syrah were collected from Qingtongxia, Ningxia Province, China (105.52° E, 38.03° N). The selected vineyard was established in 2015, all of the vines were own-rooted, with 1.0 m plant spacing and 2.0 m vine rows, and all of the agronomic practices of the vineyard followed standard management approaches. The samples were collected in two consecutive years, sampling was carried out every two weeks from 8 weeks after anthesis (WAA, pre-veraison) to harvest. During sampling, 30 vines per cultivar were randomly selected and marked, and 10 grapes were randomly selected from the upper, middle, and lower parts of each vine, for a total of 900 grapes. These were quickly frozen with liquid nitrogen, and then transported to a laboratory and stored in a −80 °C refrigerator. 

### 2.3. Determination of Basic Physicochemical Parameters 

According to the National Standard of the People’s Republic of China (GB/T 15038-2006), RS (expressed as milligrams glucose per liter) was determined with Fehling reagent, and TA (expressed as milligrams sulfuric acid per liter) was determined by sodium hydroxide titration. The berry pH was measured using a pH meter (pH-10, Sartorius, Gottingen, Germany). 

### 2.4. Extractions of Polyphenols

The extractions of polyphenols and anthocyanins were undertaken according to Xie et al. [20]. A total of 200 berries, which were frozen in a −80 °C freezer, were selected randomly from each sample, and were used to extract phenolics. Berries were peeled and skins were homogenized in liquid nitrogen with a chilled pulverizer, and skins were dried with a lyophilizer. To unify the process, 0.5 g of dried skin powder per sample was weighed and placed in 50 mL centrifuge tubes (pre-wrapped with black tape), and 10 mL of extracting solution (60% methanol, 0.1% hydrochloric acid) was added. After sonication at 30 °C and 40% power for 30 min, the samples were centrifuged at 4 °C and 10,000 rpm for 10 min. The supernatant of each sample was collected in a new 50 mL centrifuge tube with black tape. Extraction was then repeated two times, and the three supernatants were combined and stored in a −80 °C refrigerator. All extraction steps were performed under dark conditions to prevent decomposition and oxidation of phenols. 

### 2.5. Analysis of Phenolics in Grape Skin

TP content was assessed using the Folin–Ciocalteu method [21], and results are expressed as milligrams gallic acid equivalent (GAE) per gram of dry skin weight (mg GAE/g DW). TFO content was determined according to Peinado et al. [22], and results are expressed as milligrams (+)-catechin equivalent (CE) per gram of dry skin weight (mg CE/g DW). TFA was determined with p-DMACA [23], and results are expressed as milligrams rutin equivalent (RE) per gram of dry skin weight (mg RE/g DW). The TA content was estimated using the method of pH differential [24], and results are expressed as milligrams of cyanidin-3-glucoside equivalent per gram of dry skin weight (mg Cy-3-G/g DW).

### 2.6. Extractions and Determination of Individual Anthocyanins

Grapes at 8, 12, and 16 WAA of 5 cultivars were used to determine individual anthocyanins. Specific extraction steps have been described previously [20]. Three independent samples were extracted for each skin, and were stored at −40 °C until analysis. Before injection, the extracts were filtered through 0.45 µm filters (cellulose acetate and nitrocellulose, CAN).

Individual anthocyanins of extracts were analyzed using an Agilent 1100 series LC-MSD trap VL equipped with a diode array detector and a Kromasil C18 column (250 × 4.6 mm, 5 μm) (Agilent Technologies, Santa Clara, CA, USA) utilizing a binary solvent gradient, where mobile phase A was 2% formic acid in water and B was 2% formic acid in acetonitrile. The detailed LC procedures and MS conditions have been described previously report [20]. 

The elution order and retention time of the anthocyanins were compared with those of the standard, and the weights of molecular ions and fragment ions were compared with the weights of standard and reference products, allowing different anthocyanins to be identified [25,26]. Anthocyanins were quantified using malvidin-3-*O* glucoside as an external standard.

### 2.7. Quantitative Real-Time PCR

Grape skin RNA was extracted using a universal plant total RNA extraction kit (Biotech, Beijing, China), and then cDNA was synthesized by reverse transcription using an EasyScript^®^ One-Step gDNA Removal and cDNA Synthesis Super Mix Kit (Transgene, Beijing, China). A ChamQ^TM^ SYBR^®^ qPCR Master Mix (Vazyme, Beijing, China) kit was used to detect the expression levels of *VvUFGT*, *VvOMT*, *VvGST1*, *VvGST2*, *VvGST3*, *VvGST4*, *VvAM1*, and *VvAM3* [12] in skin. Quantitative real-time PCR was performed using the QuantStudio 5 Real-Time PCR System. All gene primer sequences were designed on NCBI or according to previous research [10,12] (Table S1), and primer specificity was tested by agarose gel electrophoresis and melting curve analysis. The gene expression levels were normalized to *VvActin* (Gene ID: 100246726) expression, and the relative expression levels of each gene were calculated with the 2^−ΔCT^ method, where ΔCT = CT_target_ − CT*_VvActin_*.

### 2.8. Statistical Analysis

All experiments were performed in triplicate. The data were processed using Excel 2013 and SPSS 23.0, and significant differences were compared by Duncan’s method. Figures were drawn using Origin 2018.

## 3. Results and Discussion

### 3.1. Subsection

#### 3.1.1. Reducing Sugar, Titratable Acidity, and pH

The RS content of the five grape cultivars increased from pre-veraison to harvest (Table 1), and the synthesis of sugar was more active from 8 to 12 WAA, whereas from 12 to 16 WAA, the sugar content of the five cultivars was steady. In 2018 and 2019, the RS content of Pinot Noir was the highest, at 255.11 and 240.42 g/L, respectively. The sugar content of teinturier cultivars Yan 73 and Dunkelfelder were significantly lower, and were 200–220 g/L.

The TA content decreased from 8 to 16 WAA in 2018 and 2019 (Table 1), and the content of acid decreased more rapidly from 10 to 12 WAA. Cabernet Sauvignon had the highest acid content at the harvest of the five cultivars, whereas Dunkelfelder had the lowest during the five periods of ripening. The trends of these indexes were consistent with the trend of nutrient accumulation in grapes [27]. In 2018, the pH value increased from 8 to 12 WAA in the five cultivars, and the highest pH values of the five cultivars were seen at 12 or 14 WAA. However, in 2019, the pH value increased in all five periods. The highest pH values were observed in Pinot Noir in two consecutive years, and were 3.89 and 4.62, respectively. 

At 16 WAA, the RS content of teinturier cultivars was lower than that of non-teinturier cultivars in the same area, representing a significant difference between teinturier and non-teinturier cultivars. Similar results were found in the research of Guan et al. [28]. There may be two reasons for the low sugar content of teinturier grapes. One reason is the low activity of enzymes related to sugar accumulation [29,30], sucrose-phosphate synthase, and sucrose synthase. The other main reason is that anthocyanins are formed by anthocyanidins and sugar, and the sugar was used to synthesize anthocyanins. No significant differences were found between teinturier and non-teinturier cultivars in terms of titratable acid and pH value.

The sugar-acid ratio is one of the most important indicators for evaluating the quality of wine grapes; all of the samples showed a suitable sugar-acid ratio (>25). The lowest sugar–acid ratio was found in Cabernet Sauvignon in 2018 and 2019 (29.9 and 33.5, respectively). The highest were found in Dunkelfelder (37.9, in 2018) and Pinot Noir (42.8, in 2019). Higher sugar levels in grapes have been caused by rising temperatures during recent growing seasons [31]. All samples from Qingtongxia showed good maturity and met the requirements of subsequent tests.

#### 3.1.2. Total Phenolics, Total Flavanols, and Total Flavonoids

**Figure 1:**
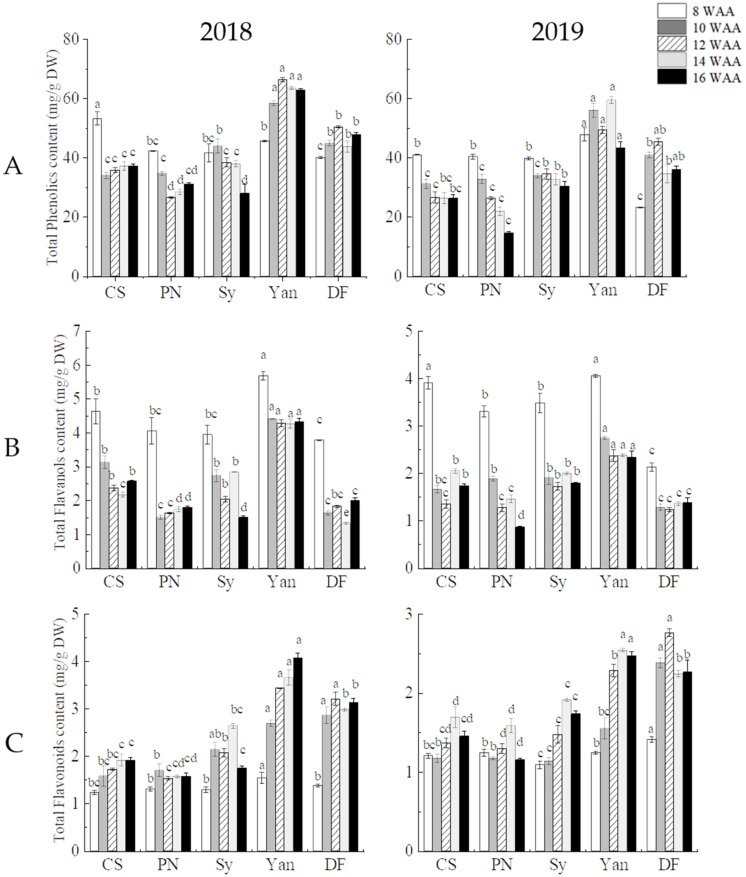
(**A**) Total phenolics, (**B**) total flavanols, and (**C**) total flavonoids of five cultivars during ripening. In the same period, different lowercase letters (a–d) in different cultivars indicate statistically significant differences at *p* < 0.05. WAA, weeks after anthesis. CS, Cabernet Sauvignon. PN, Pinot Noir. Sy, Syrah. YAN, Yan 73. DF, Dunkelfelder. The same applies below.

As shown in (Figure 1), grape cultivars varied in polyphenol content. In the five cultivars, the content of TFO increased during ripening, whereas at 14 or 16 WAA, there was a small decrease in most samples (with the exception of Yan 73 in 2018). The trend of TFA content was opposite to that of TFA, which showed a downward trend during ripening but a slight increase at 14 or 16 WAA (with the exception of Yan 73). The total phenolic content of non-teinturier cultivars generally decreased during ripening, whereas in teinturier cultivars, phenolic content first increased then decreased at 14 or 16 WAA (regression analysis was shown in Figures S1 and S2). 

At harvest, Yan 73 had the highest content of polyphenols (TP, TFA, and TFO) in 2018 and 2019, and Dunkelfelder showed the second highest content of TP and TFA. Compared with teinturier cultivars, polyphenol content of non-teinturier cultivars was lower; Pinot Noir had the lowest in 2019, whereas in 2018, Syrah and Pinot Noir had the lowest content of TP, TFA, and TFO. 

Flavanols are an important co-pigment in wine, and have an impact on wine flavor and color. It has been reported that flavanol concentrations decrease with grape ripening [32], which is in accordance with the current research.

Flavonoids are a large group of phenolic secondary metabolites that are widespread among plants [33]. The flavonoids in grape skins mainly include flavanols and anthocyanins [5]. In our research, in the five cultivars the content of flavanols showed a downward trend, whereas the content of flavonoids showed an upward trend. This may be caused by the increasing anthocyanin content, and indicated that anthocyanins are the main component of flavonoids.

TP contain a variety of phenolic substances. The biosynthesis of phenolic compounds depends largely on the genotypes of the grape cultivar rather than the environmental factors [34]. Zhu et al. [35] determined 29.12–37.72 mg/g DW of total phenolics in Cabernet Sauvignon grape skin at harvest. Samoticha et al. [17] found 32.00 and 30.72 mg/g DW of TP in Cabernet Sauvignon and Pinot Noir, respectively, which were consistent with our results. However, the TP content of teinturier cultivars was found to be higher than that of non-teinturier cultivars. This indicates that teinturier cultivars have greater potential in winemaking due to their high polyphenols. 

Furthermore, it has been confirmed that polyphenols have strong antioxidant capacity [36], so teinturier cultivars might possess a higher skin antioxidant capacity compared to other cultivars.

#### 3.1.3. Characteristics of Anthocyanins in Grape Skin

**Figure 2:**
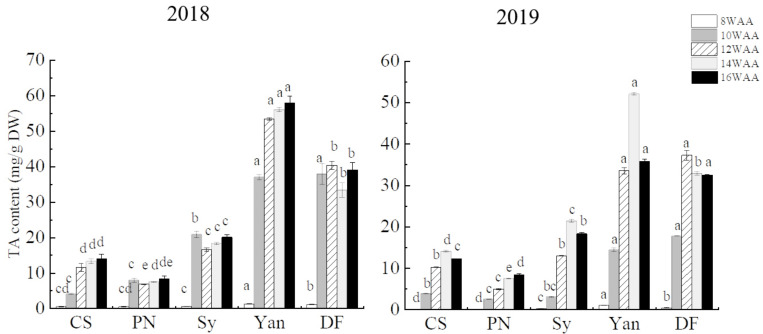
Total anthocyanins of five cultivars during ripening. Results are expressed as milligrams cyanidin-3-glucoside equivalent per gram of dry berry skin (mg Cy-3-G/g DW). In the same period, different lowercase letters (a–d) in different cultivars indicate statistically significant differences at *p* < 0.05.

Each grape variety has a unique set of anthocyanins in grape skins. Our results showed that in the five grape cultivars, the total anthocyanin content of teinturier cultivars was higher than that of non-teinturier grapes (at 16 WAA, Figure 2). Yan 73 had the highest total anthocyanin content, which was 57.87 mg/g in 2018 and 35.75 mg/g in 2019; Pinot Noir had the lowest, which was 8.43 mg/g in 2018 and 8.48 mg/g in 2019.

During ripening, the total anthocyanin content of the five cultivars showed an upward trend in 2018; however, in 2019, the anthocyanin content decreased slightly at 16 WAA. This may be due to the high temperature during 14–16 WAA. The reduction of anthocyanin accumulation due to high temperature occurs in plants not only by inhibiting the expression of anthocyanin activators and related structural genes and/or enhancing that of repressors [37,38,39], but likely also by enhancing degradation [40,41].

1. Characteristics of anthocyanin compositions in skins

Different kinds of anthocyanin compositions were detected in different periods, even in the same cultivar (Table S2). At 8 WAA, 12 kinds of anthocyanins were detected in teinturier cultivars, which was more than was detected non-teinturier cultivars. At 12 and 16 WAA, more than 16 kinds of anthocyanin were detected in four cultivars, with the exception of Pinot Noir. Thus, an active period (8–12 WAA) of anthocyanin modification was proposed. The only exception was that five basic anthocyanins, but no modified anthocyanins, were detected in Pinot Noir during all three periods. The main anthocyanins in non-teinturier cultivars were Mv and Mv-ac, whereas Mv and Pt were dominant in teinturier cultivars. All of the grape anthocyanin profiles included the established profile that was previously reported [18,42]. Dunkelfelder is also called Dornfelder, which is a German grape cultivar, for which little research has been reported. According to our results, Dunkelfelder can be classified as *V. vinifera*, which consists only of anthocyanin mono-glucosides [43]. Interestingly, Pn-ac, Mv-ac, and t-Mv-co were detected in four cultivars at 8 WAA, indicating that the acetylation modification of anthocyanins and the modification of Mv started at an earlier time.

2. Characteristics of anthocyanin types in skins

**Figure 3:**
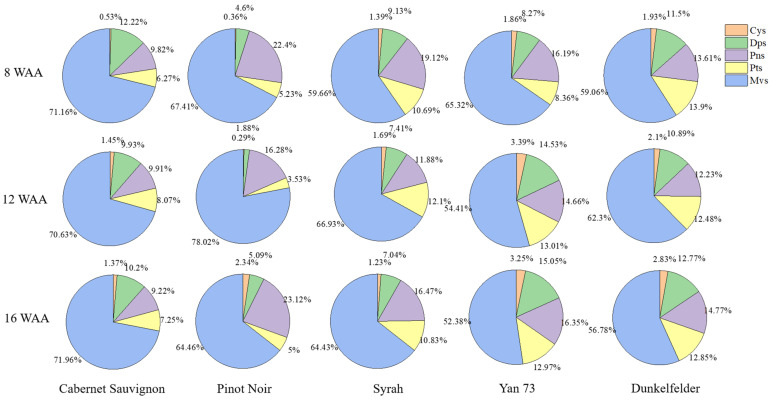
The ratio of anthocyanin components.

The ratio of anthocyanin components during three periods showed little change; thus, 16 WAA was taken as an example (data with a significance test is shown in Table S3). At 16 WAA, malvidins were the most frequent anthocyanins in all cultivars (Figure 3). Among the cultivars, non-teinturier cultivars had higher content (>64%) and content of teinturier cultivars was lower (<57%). Similar results were also found in the studies of Guan et al. [44] and Castillo-Munoz et al. [45]. This may be a characteristic of teinturier cultivars. The second most common anthocyanin component in the five cultivars varied; delphinidins were found in Cabernet Sauvignon, whereas peonidins were found in the other four cultivars. Cyanidins were the least-common anthocyanin component in each of the five cultivars. Furthermore, in contrast to peonidins and petunidins, the ratios of delphinidins in teinturier cultivars were significantly higher than those in non-teinturier cultivars, and the ratios of malvidins were the lowest in teinturier cultivars. Thus, the activity of 3′ OMT in the teinturier grape was similar to that of the other three cultivars, whereas the activity of 5′ OMT was significantly lower, according to the calculation method of Mattivi et al. [46]. However, to the best of our knowledge, no genes specifically encoding the 3′ OMT and 5′ OMT have been identified [7]. Our results assumed that there were two pairs of genes that determined the enzyme activity of 3′ OMT and 5′ OMT. A similar case was reported in petunia (Petunia hybrida), in which Mt and Mf were responsible for the methylation of the anthocyanin molecule at the 3′ and 5′ positions, respectively. When Mt was dominant, 3′-O-methylated anthocyanins (peonidin or petunidin) mainly accumulated in the flower, whereas the 3′,5′-O-methylated malvidin accumulated as the main pigment only when Mf was dominant [47]. Our results suggest that two pairs of genes control the methylated anthocyanins in grapes. As in petunia, a Mf-like OMT was less dominant in teinturier cultivars than in non-teinturier cultivars.

3. Characteristics of anthocyanin modification in skins

As shown in (Table 2), all the samples revealed a high proportion (above 80%) of methylated anthocyanins. Non-teinturier cultivars showed a higher proportion of methylated anthocyanins than teinturier cultivars. In grapes, anthocyanins can be modified not only by O-methyltransferases, but also by acyltransferases, which produce 3-*O*-acetyl-, 3-*O*-coumaroyl-, and 3-*O*-caffeoyl-monoglucosides by attaching acyl groups to the C6″ position of the Glc moiety [48]. Acylated anthocyanins are more stable compared with their nonacylated counterparts, most likely due to increased intramolecular stacking [49,50]. The percentage of acylated anthocyanin of the five grape cultivars differed. The highest proportion of acylated anthocyanins was also shown in non-teinturier cultivars (above 40% at 16 WAA, with the exception of Pinot Noir), whereas the lowest was shown in teinturier cultivars (29–30% at 16 WAA). No acylated anthocyanins were detected in Pinot Noir. Among three kinds of acylated anthocyanins, the ratio of caffeoylated anthocyanins was the lowest (<2.5% in all samples). More acetylated anthocyanins than coumarylated anthocyanins were found in Cabernet Sauvignon and Yan 73, whereas the opposite result was found for Syrah, and the percentages of acetylated and coumarylated anthocyanins in Dunkelfelder were both 14%. Our results revealed that despite the large number of anthocyanins found in Yan 73 and Dunkelfelder, the stability of these cultivars is not as good as that of non-teinturier cultivars.

4. Principal component analysis (PCA) of different cultivars

**Figure 4:**
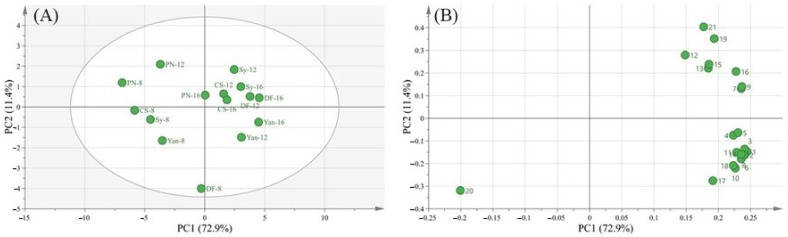
Principal component analysis (PCA) of different cultivars during ripening. In score plot (**A**), the number behind the cultivars means the weeks after anthesis. In loading plot (**B**), 1, delphinidin−3−glucoside. 2, cyanidin−3−glucoside. 3, petunidin−3−glucoside. 4, peonidin−3−glucoside. 5, malvidin−3−glucoside. 6, delphinidin−3−glu acetate. 7, cyanidin−3−glu acetate. 8, petunidin−3−glu acetate. 9, delphinidin−3−glu coumarate. 10, peonidin−3−glu acetate. 11, malvidin−3−glu acetate. 12, cyanidin−3−glu coumarate. 13, malvidin−3−glu caffeate. 14, petunidin−3−glu coumarate. 15, *cis*−peonidin−3−glu coumarate, 16, *cis*−malvidin−3−glu coumarate. 17, *trans*−peonidin−3−glu coumarate. 18, *trans*−malvidin−3−glu coumarate. 19, reducing sugar. 20, titratable acid. 21, pH value

To further investigate the differences between cultivars, a principal component analysis was performed. The score plot (Figure 4A) showed that PC1, which explained 72.9% of the total variance, mainly distinguished the development stage, cultivars in 8 WAA were located in the negative part of PC1, whereas grapes in 12 or 16 WAA were located in the positive part. In the loading plot (Figure 4B), PC1 was positively correlated with the concentration of RS, pH, and individual anthocyanins, and negatively correlated with TA because of their different trends of accumulating during ripening. Most teinturier cultivars were separated from non-teinturier cultivars by PC2, which explained 11.4% of the total variance. RS, pH, and TA were the components that contribute more to PC2.

#### 3.1.4. Gene Relative Expression Related to Anthocyanin Biosynthesis, Modification, and Transport

**Figure 5:**
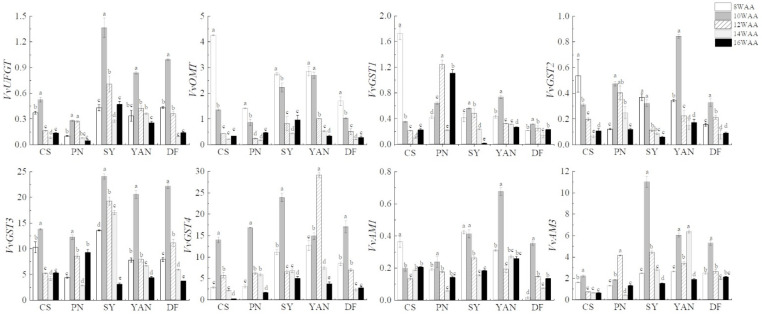
Relative gene expression of five cultivars during ripening in 2018. Different lowercase letters (a-e) in the same cultivar denote statistically significant differences at *p* < 0.05.

**Figure 6:**
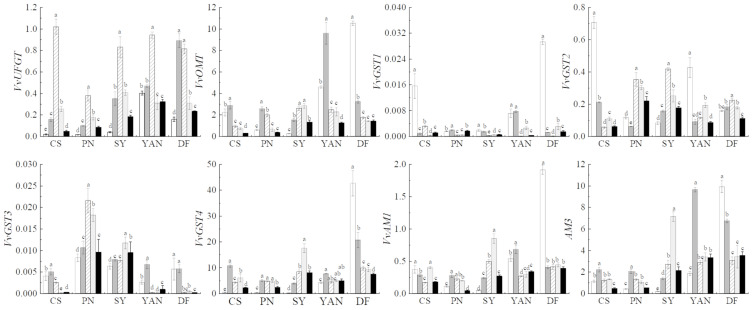
Relative gene expression of five cultivars during ripening in 2019. Different lowercase letters (a-e) in the same cultivar denote statistically significant differences at *p* < 0.05.

The changes in gene expression are shown in (Figure 5). The results showed that the expression of *VvUFGT* in all grape cultivars increased significantly from 8 to 10 WAA (2018) or 12 WAA (2019), and then decreased until harvest. It has been reported that the *VvUFGT* gene expression was detected only after the coloring stage began [6]. Combined with our results showing extremely low anthocyanin content at 8 WAA, this indicates anthocyanin synthesis might start at 8 WAA or slightly earlier. Furthermore, cultivars with higher *VvUFGT* expression also had higher anthocyanin content. The expression trends of *VvOMT* in the five cultivars were the same in 2018, showing a significantly high level at 8 and 10 WAA, and then decreasing during the final three periods. In 2019 (Figure 6), the expression level of four cultivars (with the exception of Dunkelfelder) first increased and then decreased. The differences between *VvOMT* and *VvUFGT* in the first period indicate that *VvOMT* was not only responsible for the methylation of anthocyanin, but also for other metabolites [51]. These differences also indicate the methylation of flavanols started at an earlier time. *VvGSTs* and *VvAMs* are genes related to anthocyanin transport, and both reached their highest values at 8, 10, or 12 WAA in different cultivars; that is, during the period when anthocyanin content increased rapidly. In 2018 and 2019, *VvGST4* and *VvAM3* had a higher expression level than other transport genes, which indicates that they played an important role in anthocyanin transport [10]. It has been reported that AMs were correlated with the transport of acylated anthocyanin [12]. Our results do not support this conclusion.

#### 3.1.5. Cluster Analysis of Anthocyanin Accumulation and Gene Expression 

**Figure 7:**
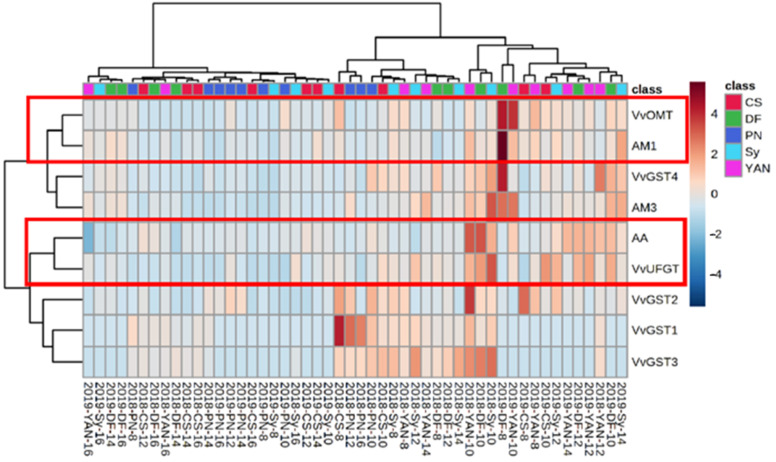
Cluster analysis heatmap of anthocyanin accumulation and gene expression of anthocyanin synthesis, modification, and transport. The sample mark below means: year–cultivar–weeks after anthesis.

To identify the regular pattern of anthocyanin accumulation (AA) and gene expression of anthocyanin synthesis, modification, and transport, cluster analysis was performed using the two-year anthocyanin gene expression and anthocyanin accumulation of the five cultivars. The results of cluster analysis showed a clear grouping pattern (Figure 7).

AA and *VvUFGT* were clustered together, indicating that *VvUFGT* regulates the accumulation of anthocyanins, which is also consistent with the current research conclusions [52]. 

*VvOMT* was clustered with *VvAM1*, which indicates that *VvAM1* is related to the methylated anthocyanins. In combination with the transport function of MATE protein, we assume that *VvAM1* is responsible for the transport of methylated anthocyanins. This result seems to be contrary to the conclusion of Gomez et al. [12] that *VvAMs* are responsible for the transport of acylated anthocyanins. However, existing research into the transport gene has mainly been conducted in vitro, and differences between in vivo and in vitro studies are not clear. Furthermore, no conclusion has been drawn about whether the genes provide the same function in different cultivars. Only by combining the trend of gene expressions with methylated anthocyanins’ accumulation during ripening can further conclusions be drawn.

## 4. Conclusions

In this study, the changes and differences in grape quality, polyphenol content, anthocyanin profile, and related gene expression between teinturier and non-teinturier cultivars during ripening were explored. The results show that the reduced sugar content of teinturier cultivars was lower than that of non-teinturier cultivars. However, the sugar–acid ratios of the five cultivars were all above 25 and were sufficiently high for wine making. At harvest, the polyphenol content (TP, TFA, TFO, TA) of Yan 73 was the highest in the five cultivars, and Dunkelfelder was the second highest (with the exception of TFA). 

The anthocyanin accumulation profiles showed that 17 or 18 kinds of individual anthocyanin were detected in teinturier cultivars, which is consistent with Cabernet Sauvignon and Syrah. However, the total content of individual anthocyanins in teinturier cultivars was significantly higher than that in non-teinturier cultivars. Analysis of the types of individual anthocyanins showed that the ratios of methylated and acylated anthocyanins proportions in teinturier cultivars are lower than those of Cabernet Sauvignon and Syrah, which indicates the anthocyanin stability of teinturier cultivars is not as good as that of non-teinturier cultivars. 

The principal component analysis distinguishes the development stage of cultivars by PC1, and separated teinturier and non-teinturier cultivars by PC2. Cluster analysis of anthocyanin accumulation and eight genes related to anthocyanin synthesis, modification, and transport, indicated that *VvUFGT* was related to anthocyanin synthesis and *VvAM1* may be related to the transport of methylated anthocyanins. Overall, these results provide a reference for studies of anthocyanin synthesis, modification, and transport in teinturier cultivars.

## Figures and Tables

**Table 1:** Reducing sugar, titratable acid, and pH of five cultivars during ripening.

	Period (WAA)	Cabernet Sauvignon			Pinot Noir			Syrah			Yan73			Dunkelfelder		
		RS	TA	pH	RS	TA	pH	RS	TA	pH	RS	TA	pH	RS	TA	pH
2018	8	35.02 ± 9.90 dC	24.21 ± 1.90 aA	2.55 ± 0.03 dB	54.02 ± 2.83 dB	21.22 ± 0.05 aB	2.72 ± 0.05 dA	69.03 ± 1.41 dA	20.94 ± 0.00 aBC	2.69 ± 0.02 eA	57.03 ± 0.82eB	25.50 ± 0.17 aA	2.42 ± 0.00 dC	38.04 ± 1.05 cC	23.48 ± 1.12 aAB	2.51 ± 0.05 dB
	10	139.56 ± 0.71 cD	19.00 ± 0.71 bA	2.87 ± 0.03 cC	192.08 ± 2.83 cA	11.41 ± 0.05 bC	3.28 ± 0.00 cB	159.07 ± 4.24 cC	14.14 ± 0.10 bBC	3.04 ± 0.03 dBC	160.07 ± 0.82dC	16.40 ± 0.00 bB	2.78 ± 0.00 cC	174.08 ± 2.83 bB	9.84 ± 0.00 bD	3.70 ± 0.03 cA
	12	176.08 ± 2.83 bD	12.54 ± 1.83 cA	3.84 ± 0.00 aB	185.08 ± 1.41 cC	8.40 ± 0.10 cC	3.96 ± 0.04 aA	224.10 ± 2.83 bA	10.00 ± 0.05 cB	3.76 ± 0.02 cBC	178.08 ± 1.63cD	9.64 ± 0.03 cB	3.53 ± 0.05 aC	210.59 ± 3.54 aB	7.84 ± 0.13 cCD	3.96 ± 0.01 aA
	14	220.10 ± 2.83 aB	8.70 ± 0.83 dA	3.60 ± 0.03 bC	206.09 ± 11.32 bC	8.10 ± 0.14 cAB	3.82 ± 0.04 bB	240.61 ± 2.12 aA	8.22 ± 0.05 dA	4.08 ± 0.03 aA	228.10 ± 0.00aAB	7.92 ± 0.08 dAB	3.42 ± 0.02 bD	210.59 ± 2.12 aBC	6.02 ± 0.13 dC	3.86 ± 0.03 bB
	16	223.10 ± 0.00aC	7.46 ± 0.00 eA	3.72 ± 0.03 bB	255.11 ± 1.41 aA	6.74 ± 0.09 dB	3.89 ± 0.02 aA	241.61 ± 0.71 aB	6.56 ± 0.09 eB	3.83 ± 0.03 bA	211.59 ± 2.04bD	6.56 ± 0.05 eB	3.83 ± 0.02 bA	216.10 ± 2.83 aD	5.70 ± 0.05 dC	3.85 ± 0.03 bA
2019	8	16.47 ± 5.04 dD	29.79 ± 0.42 aA	2.58 ± 0.02 dC	46.02 ± 1.00 eA	25.88 ± 2.58 bC	2.71 ± 0.01 cB	32.01 ± 14.74 eB	26.71 ± 0.33 aBC	2.58 ± 0.01 cC	19.51 ± 0.00eCD	30.32 ± 0.27 aA	2.52 ± 0.01 dC	22.68 ± 4.04 cC	27.36 ± 0.18 aB	2.89 ± 0.03 cA
	10	119.65 ± 3.47 cA	25.06 ± 0.06 bA	3.37 ± 0.01 cC	98.71 ± 4.17 dB	23.37 ± 0.20 aB	3.60 ± 0.01 bB	77.37 ± 2.52 dD	23.26 ± 0.47 aB	3.33 ± 0.02 bC	74.13 ± 2.65dD	22.51 ± 0.29 bB	3.24 ± 0.03 cCD	83.37 ± 0.58 bC	25.96 ± 0.05 bA	3.83 ± 0.01 bA
	12	177.74 ± 2.52 bA	12.18 ± 0.38 cB	3.34 ± 0.01 cB	136.06 ± 2.65 cD	7.84 ± 0.05 cD	3.71 ± 0.01 bA	142.40 ± 4.04 cCD	11.42 ± 0.13 bC	3.36 ± 0.01 bB	150.00 ± 3.22cC	14.90 ± 0.05 cA	3.26 ± 0.01 cC	167.02 ± 2.00 aB	10.82 ± 0.18 dC	3.76 ± 0.01 bA
	14	226.30 ± 2.00 aA	9.98 ± 0.29 dB	4.04 ± 0.02 bB	172.74 ± 4.62 bD	6.24 ± 0.09 dC	4.41 ± 0.01 aA	151.73 ± 0.58 bE	9.78 ± 0.05 cB	4.18 ± 0.02 aB	184.25 ± 1.53bC	11.56 ± 0.09 dA	3.89 ± 0.01 bC	211.13 ± 0.58 B	7.24 ± 0.05 cC	4.09 ± 0.02 aB
	16	230.26 ± 5.13 aB	6.88 ± 0.04 eA	4.36 ± 0.02 aAB	240.42 ± 1.53 aA	5.62 ± 0.05 dB	4.62 ± 0.01 aA	237.42 ± 0.58 aA	5.76 ± 0.09 dB	4.13 ± 0.02 aB	202.02 ± 1.16aD	5.86 ± 0.05 dB	4.06 ± 0.01 aC	219.36 ± 2.31 aC	5.23 ± 0.00 dC	4.19 ± 0.01 aB

Note: WAA, weeks after anthesis. RS, reducing sugar, g/L. TA, titratable acid g/L. Results are expressed as means ± SD of three biological replicates (*n* = 3). The same applies below. Different lowercase letters in the same column, and for each year separately, demonstrate that the difference was significant (*p* < 0.05). Capital letters in the same row and same parameters demonstrate that the difference was significant (*p* < 0.05).

**Table 2:** The content and proportion of anthocyanin modifications in grape skins of different cultivars.

Cultivars	Period (WAA)	Methylation		Acetylation		Caffeination		Coumarylation		Acylation	
		Content (mg/kg)	Proportion (%)	Content (mg/kg)	Proportion	Content (mg/kg)	Proportion	Content (mg/kg)	Proportion	Content (mg/kg)	Proportion
Cabernet Sauvignon	8	255.91 ± 6.24	87.25	93.31 ± 2.56	31.81	nd	0	15.57 ± 0.07	5.31	108.88 ± 3.15	37.12
	12	11,085.23 ± 269.37	88.61	4164.79 ± 110.78	33.29	116.76 ± 2.55	0.93	1397.69 ± 9.22	11.17	5679.23 ± 119.83	45.40
	16	15,261.50 ± 74.78	88.44	5415.56 ± 102.35	31.38	nd	0	2062.32 ± 6.39	11.95	7477.89 ± 164.51	43.33
Pinot Noir	8	271.83 ± 1.71	95.04	nd	0	nd	0	nd	0	nd	0
	12	6553.20 ± 43.25	97.83	nd	0	nd	0	nd	0	nd	0
	16	7934.72 ± 44.43	92.57	nd	0	nd	0	nd	0	nd	0
Syrah	8	29.03 ± 0.46	89.47	3.96 ± 0.09	12.20	nd	0	5.28 ± 0.14	16.28	9.24 ± 0.13	28.48
	12	780.58 ± 19.05	90.90	194.19 ± 2.89	22.61	18.81 ± 0.35	2.19	269.26 ± 6.06	31.36	482.26 ± 8.05	56.16
	16	1003.43 ± 22.08	91.73	235.23 ± 2.82	21.50	11.63 ± 0.11	1.06	332.50 ± 8.38	30.39	579.36 ± 11.76	52.96
Yan 73	8	1470.63 ± 12.50	89.87	287.72 ± 7.80	17.58	nd	0	102.33 ± 1.32	6.25	390.05 ± 3.55	23.84
	12	47,396.15 ± 1346.05	82.09	12,522.05 ± 40.07	21.69	nd	0	5045.41 ± 101.41	8.74	17,567.46 ± 176.00	30.43
	16	81,143.90 ± 1420.02	81.69	19,201.10 ± 499.23	19.33	279.13 ± 2.82	0.28	7232.52118.61	7.28	26,712.75 ± 323.22	26.89
Dunkelfelder	8	1280.15 ± 4.99	86.57	359.76 ± 1.26	24.33	16.39 ± 0.31	1.11	328.11 ± 6.20	22.19	704.26 ± 19.44	47.62
	12	38,467.77 ± 411.61	87.01	6506.72 ± 35.14	14.72	276.10 ± 4.50	0.62	6573.00 ± 137.38	14.87	13,355.82 ± 280.47	30.21
	16	60,868.71 ± 499.12	84.40	9498.26 ± 210.86	13.17	367.66 ± 4.01	0.51	8279.74 ± 248.39	11.48	18,145.66 ± 68.95	25.16

Note: ‘nd’ means not detected.

## Data Availability

Not applicable.

## Supplementary Materials

The following are available online at https://www.mdpi.com/article/10.3390/foods10051073/s1, Table S1: Specific primers used for qRT-PCR, Table S2: The identification of anthocyanin in 5 cultivars, Table S3: Ratio of anthocyanin components, Figure S1: Regression analysis of TFA content, Figure S2: Regression analysis of TFO content.

## Abbreviations

TA	titratable acid.
RS	reducing sugar.
HPLC	high-performance liquid chromatography.
WAA	weeks after anthesis.
TP	total phenolics.
TFA	total flavanols.
TFO	total flavonoids.
TA	total anthocyanins.
Mv	malvidin−3−glucoside.
Mv−ac	malvidin−3−glu acetate.
Pt	petunidin−3−glucoside.
Pn−ac	peonidin−3−glu acetate.
t−Mv−co	*trans*-malvidin−3−glu coumarate.
