# Supplementary material for: Differences in Anthocyanin Accumulation Profiles between Teinturier and Non-Teinturier Cultivars during Ripening
Source: Foods. 2021 May 12;10(5):1073. doi: 10.3390/foods10051073 (PMC8151246; doi:10.3390/foods10051073)
Supplement: Supplementary file 1 [file foods-10-01073-s001.zip › foods-1192691-supplementary.pdf]

Table S1. Specific primers used for qRT-PCR.

Gene	Primer（5′→3′）	
VvActin	Forward	CTTGCATCCCTCAGCACCTT
	Reverse	TCCTGTGGACAATGGATGGA
VvOMT	Forward	CCATAAGCAAACCCTAAACC
	Reverse	TGAACAAATTCTTGGCATCA
VvUFGT	Forward	GGGATGGTAATGGCTGTGG
	Reverse	ACATGGGTGGAGAGTGAGTT
VvGST1	Forward	CCAAAGAGCAAAAGCCAAGT
	Reverse	TGTCCAGAAAACCCAAAGTC
VvGST2	Forward	CCCAACCCCTGAAAATCTTGCT
	Reverse	AGCGACAACTCGCATCACTG
VvGST3	Forward	GTGGAACAATGGCTGGAGGT
	Reverse	CACTTTCGCATCTGGGGGAA
VvGST4	Forward	CCTACCTCGAATGGGTGAGC
	Reverse	GTAACCAAGTGCGCCATTCC
AM1	Forward	TGCTTTTGTGATTTTGTTAGAGG
	Reverse	CCCTTCCCCGATTGAGAGTA
AM3	Forward	TGCTTTTGTGATTTTGTTAGAGG
	Reverse	CCCTTCCCCGATTGAGAGTA

Table S2. The identification of anthocyanin in 5 cultivars (mg/g DW grape skin).

	**Cabernet Sauvignon**			**Pinot Noir**			**Syrah**			Yan 73			Dunkelfelder		
	**8WAA**	**12WAA**	**16WAA**	**8WAA**	**12WAA**	**16WAA**	**8WAA**	**12WAA**	**16WAA**	8WAA	12WAA	16WAA	8WAA	12WAA	16WAA
Dp	32.83 ± 0.05	909.58 ± 1.02	1276.06 ± 25.74	13.15 ± 0.00	125.84 ± 0.03	358.86 ± 0.13	59.26 ± 0.10	941.33 ± 047	1151.80 ± 5.40	123.13 ± 0.08	6400.06 ± 2.31	11717.82 ± 10.76	107.08 ± 1.10	3906.72 ± 0.93	7589.28 ± 3.54
Cy	1.54 ± 0.05	132.18 ± 0.10	160.72 ± 0.03	1.03 ± 0.00	19.71 ± 0.46	161.20 ± 0.52	9.05 ± 0.10	167.37 ± 14.67	178.10 ± 0.57	30.40 ± 0.03	1509.25 ± 0.51	2578.87 ± 39.19	28.47 ± 1.14	772.55 ± 0.08	1777.51 ± 1.97
Pt	18.38 ± 0.10	667.00 ± 0.11	875.49 ± 0.30	14.97 ± 0.00	236.17 ± 0.95	399.08 ± 0.57	60.11 ± 0.13	1102.06 ± 1.19	1336.19 ± 7.43	117.89 ± 0.18	5316.68 ± 2.82	9600.74 ± 62.08	148.85 ± 1.40	4029.92 ± 1.38	7195.35 ± 7.33
Pn	19.26 ± 0.00	668.01 ± 0.17	813.28 ± 0.64	64.05 ± 0.00	1090.68 ± 0.66	1841.33 ± 0.32	71.15 ± 0.39	893.85 ± 0.70	1291.49 ± 6.86	211.92 ± 0.26	5981.98 ± 32.68	12101.42 ± 34.38	130.73 ± 2.40	3518.71 ± 1.12	7701.94 ± 10.80
Mv	112.40 ± 0.13	4453.52 ± 0.21	6653.84 ± 4.70	192.80 ± 0.00	5140.69 ± 20.23	4820.65 ± 2.54	264.46 ± 0.26	4334.14 ± 2.33	6333.70 ± 25.35	763.03 ± 0.40	20963.90 ± 99.55	36615.84 ± 18.28	595.57 ± 3.39	18625.23 ± 3.75	29708.69 ± 26.05
Dp-ac	3.02 ± 0.18	250.43 ± 0.13	317.26 ± 0.72	nd	nd	nd	nd	259.61 ± 0.21	256.20 ± 0.05	12.26 ± 0.05	1549.749 ± 19.55	2478.50 ± 9.60	23.05 ± 1.76	559.62 ± 0.21	1090.78 ± 7.69
Cy-ac	nd	35.16 ± 0.05	75.15 ± 3.91	nd	nd	nd	nd	59.64 ± 1.29	68.28 ± 2.37	nd	312.56 ± 8.52	653.09 ± 1.46	nd	127.83 ± 0.05	214.44 ± 1.60
Pt-ac	nd	269.32 ± 0.93	329.31 ± 1.29	nd	nd	nd	nd	389.367 ± 0.26	407.91 ± 9.17	15.98 ± 0.15	1622.33 ± 14.85	2521.52 ± 4.68	27.97 ± 1.19	717.30 ± 1.61	1103.31 ± 5.80
Dp-co	nd	82.66 ± 1.03	166.59 ± 5.49	nd	nd	nd	nd	71.65 ± 3.99	132.37 ± 17.87	nd	437.91 ± 24.13	755.25 ± 4.94	nd	347.29 ± 1.60	529.90 ± 17.16
Pn-ac	9.55 ± 1.34	396.97 ± 0.59	467.46 ± 4.89	nd	nd	nd	26.90 ± 0.15	969.19 ± 2.29	1032.02 ± 16.18	42.23 ± 0.10	1838.86 ± 24.49	3028.94 ± 10.78	46.05 ± 0.05	1189.10 ± 0.66	1818.99 ± 1.91
Mv-ac	80.74 ± 2.76	3212.91 ± 3.66	4226.38 ± 4.45	nd	nd	nd	52.26 ± 0.77	2205.99 ± 0.85	2940.27 ± 19.64	217.25 ± 0.54	7064.89 ± 11.84	10519.05 ± 14.23	131.90 ± 1.12	3912.87 ± 0.23	5270.74 ± 5.76
Cy-co	nd	14.28 ± 0.52	nd	nd	nd	nd	nd	62.60 ± 0.62	23.02 ± 0.59	nd	nd	nd	nd	27.18 ± 1.31	47.81 ± 0.44
Mv-ca	nd	116.76 ± 0.28	nd	nd	nd	nd	nd	376.21 ± 1.06	232.54 ± 0.31	nd	nd	279.13 ± 3.12	nd	276.10 ± 1.83	367.66 ± 4.89
Pt-co	nd	73.48 ± 0.28	46.45 ± 0.41	nd	nd	nd	9.23 ± 0.05	586.84 ± 0.66	624.89 ± 1.57	2.93 ± 0.05	574.15 ± 0.17	756.13 ± 1.25	28.79 ± 0.03	771.59 ± 0.87	969.71 ± 3.52
c-Pn-co	nd	1.35 ± 0.10	102.50 ± 4.71	nd	nd	nd	nd	86.48 ± 0.08	46.67 ± 0.23	nd	nd	17.28 ± 3.94	nd	16.58 ± 1.31	33.11 ± 0.28
c-Mv-co	nd	46.99 ± 0.08	483.39 ± 1.67	nd	nd	nd	nd	1195.09 ± 3.09	146.00 ± 0.10	nd	71.21 ± 0.08	97.01 ± 7.29	nd	93.48 ± 0.90	123.25 ± 1.11
t-Pn-co	nd	173.91 ± 0.03	207.85 ± 0.23	nd	nd	nd	26.01 ± 0.41	nd	1233.82 ± 7.18	10.81 ± 0.15	644.22 ± 0.23	1088.63 ± 7.96	24.47 ± 0.15	682.46 ± 0.23	1094.36 ± 4.09
t-Mv-co	15.57 ± 0.49	1005.02 ± 0.15	1455.55 ± 1.14	nd	nd	nd	70.39 ± 0.33	3382.47 ± 0.49	4443.15 ± 15.89	88.59 ± 0.21	3317.93 ± 1.04	4518.22 ± 2.03	145.82 ± 1.04	4634.41 ± 3.41	5481.59 ± 1.27
Total anthocyanins	293.30 ± 4.17	12509.52 ± 2.25	17257.28 ± 30.15	286.01 ± 0.00	6613.09 ± 10.62	7581.12 ± 12.83	648.82 ± 1.62	17173.88 ± 7.50	21878.42 ± 135.94	1636.42 ± 0.81	57739.33 ± 78.30	99327.44 ± 158.01	1438.75 ± 10.39	44208.97 ± 14.61	72118.42 ± 56.08

Note: ‘nd’ means not detected. Dp, delphinidin-3-glucoside. Cy, cyanidin-3-glucoside. Pt, petunidin-3-glucoside. Pn, peonidin-3-glucoside. Mv, malvidin-3-glucoside. Dp-ac, delphinidin-3-glu acetate. Cy-ac, cyanidin -3-glu acetate. Pt-ac, petunidin-3-glu acetate. Dp-co, delphinidin-3-glu coumarate. Pn-ac, peonidin-3-glu acetate. Mv-ac, malvidin-3-glu acetate. Cy-co, cyanidin -3-glu coumarate. Mv-ca, malvidin-3-glu caffeate. Pt-co, petunidin-3-glu coumarate. c-Pn-co, cis-peonidin-3-glu coumarate, c-Mv-co, cis-malvidin-3-glu coumarate. t-Pn-co, trans-peonidin-3-glu coumarate. t-Mv-co, trans-malvidin-3-glu coumarate.

Table 3. Ratio of anthocyanin components.

Cultivars	WAA	Cyanidins	Delphinidins	Peonidins	Petunidins	Malvidins
Cabernet Sauvignon	8	1.54 ± 0.84eD	35.85 ± 0.49bC	28.81 ± 0.69cD	18.38 ± 0.09dD	208.72 ± 5.22aC
	12	181.62 ± 6.25cC	1242.67 ± 15.35bC	1240.23 ± 8.68bD	1009.8 ± 24.24bD	8835.2 ± 309.23aBC
	16	235.88 ± 3.25dC	1759.91 ± 18.88bC	1591.09 ± 12.73bcDE	1251.25 ± 55.06cD	12419.16 ± 149.03aC
Pinot Noir	8	1.03 ± 0.26dD	13.15 ± 0.65cD	64.05 ± 2.56bC	14.97 ± 0.19cD	192.8 ± 9.64aC
	12	19.71 ± 0.95eD	125.84 ± 2.85dD	1090.68 ± 5.45bD	236.17 ± 11.34cE	5226.35 ± 109.75aC
	16	200.65 ± 5.40dCD	436.00 ± 17.4cD	1981.67 ± 11.89bD	428.27 ± 6.42cE	5524.78143.64aD
Syrah	8	9.05 ± 0.28dC	59.26 ± 3.28cB	124.06 ± 3.85bB	69.34 ± 2.77cC	387.11 ± 1.94aB
	12	289.61 ± 3.74dC	1272.59 ± 18.82cC	2039.52 ± 93.82bC	2078.27 ± 85.21bC	11493.9 ± 218.38aB
	16	269.40 ± 3.90eC	1540.36 ± 13.90dC	3604.01 ± 25.23bC	2368.99 ± 14.21cC	14095.65 ± 521.54aC
Yan 73	8	30.40 ± 0.85dA	135.39 ± 1.28cA	264.95 ± 3.71bA	136.8 ± 5.06cB	1068.87 ± 28.86aA
	12	1955.48 ± 23.36cA	8387.7 ± 51.00bA	8465.06 ± 33.86bA	7513.16 ± 37.57bA	31417.93 ± 1162.46aA
	16	3231.97 ± 33.42dA	14951.57 ± 128.84bcA	16236.26 ± 204.94bA	12878.39 ± 489.38cA	52029.25 ± 1352.76aA
Dunkelfelder	8	24.94 ± 1.60cB	146.91 ± 3.68bA	235.31 ± 3.47bA	183.72 ± 4.29bA	882.82 ± 22.69aA
	12	927.56 ± 5.60cB	4813.64 ± 25.12bB	5406.86 ± 16.22bB	5518.81 ± 11.04bB	27542.1 ± 936.43aA
	16	2039.76 ± 11.65cB	9209.96 ± 84.39bB	10648.4 ± 95.85bB	9268.37 ± 185.37bB	40951.93 ± 1883.79aB

Note: Results were expressed as means ± SD of three biological replicates for (n = 3). Different lowercase letters in the same line demonstrated that the difference was significant ($p < 0.05$). Capital letters in the same row and same period demonstrated that the difference was significant ($p < 0.05$).

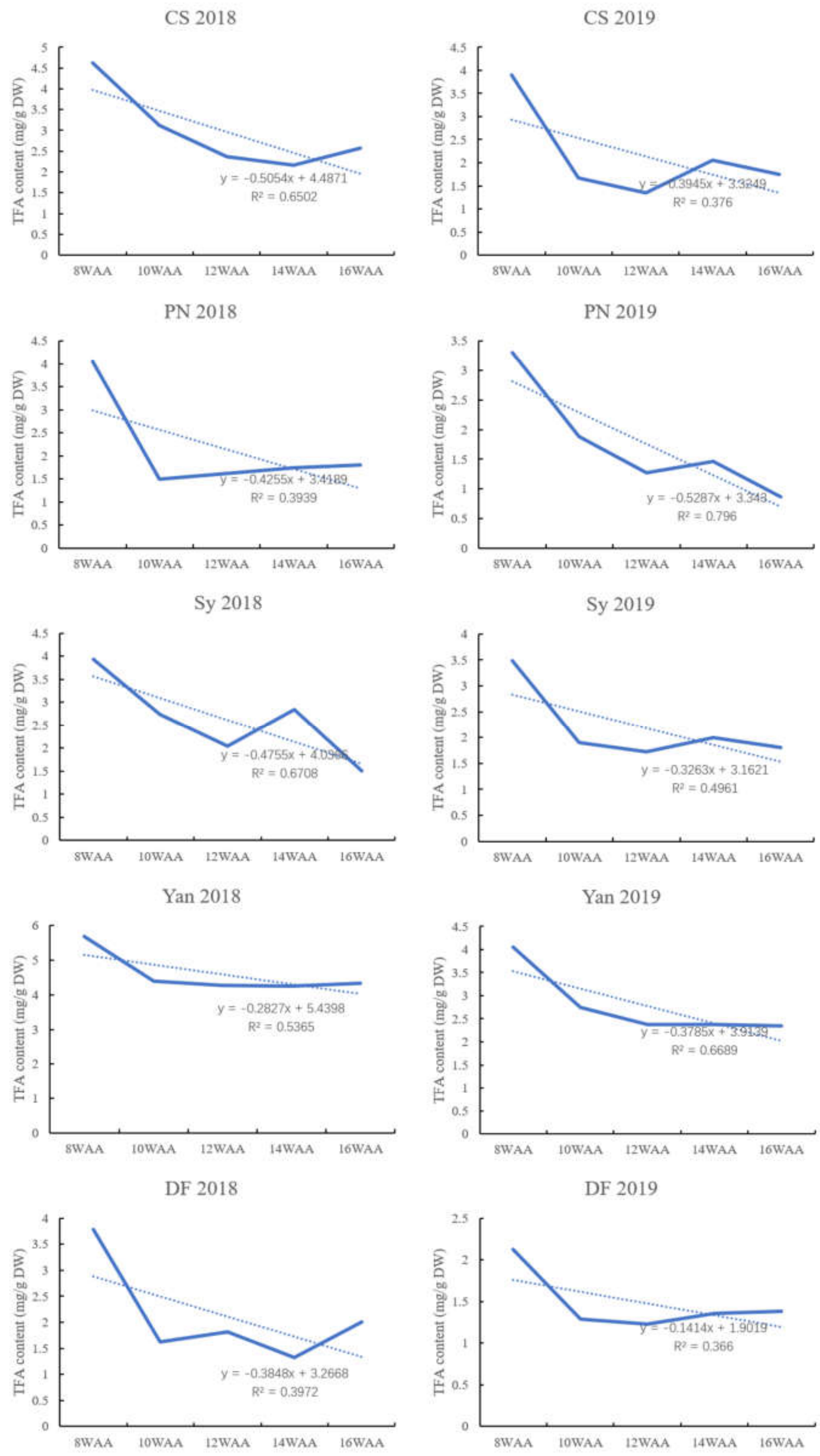

Figure S1 Regression analysis of TFA content.

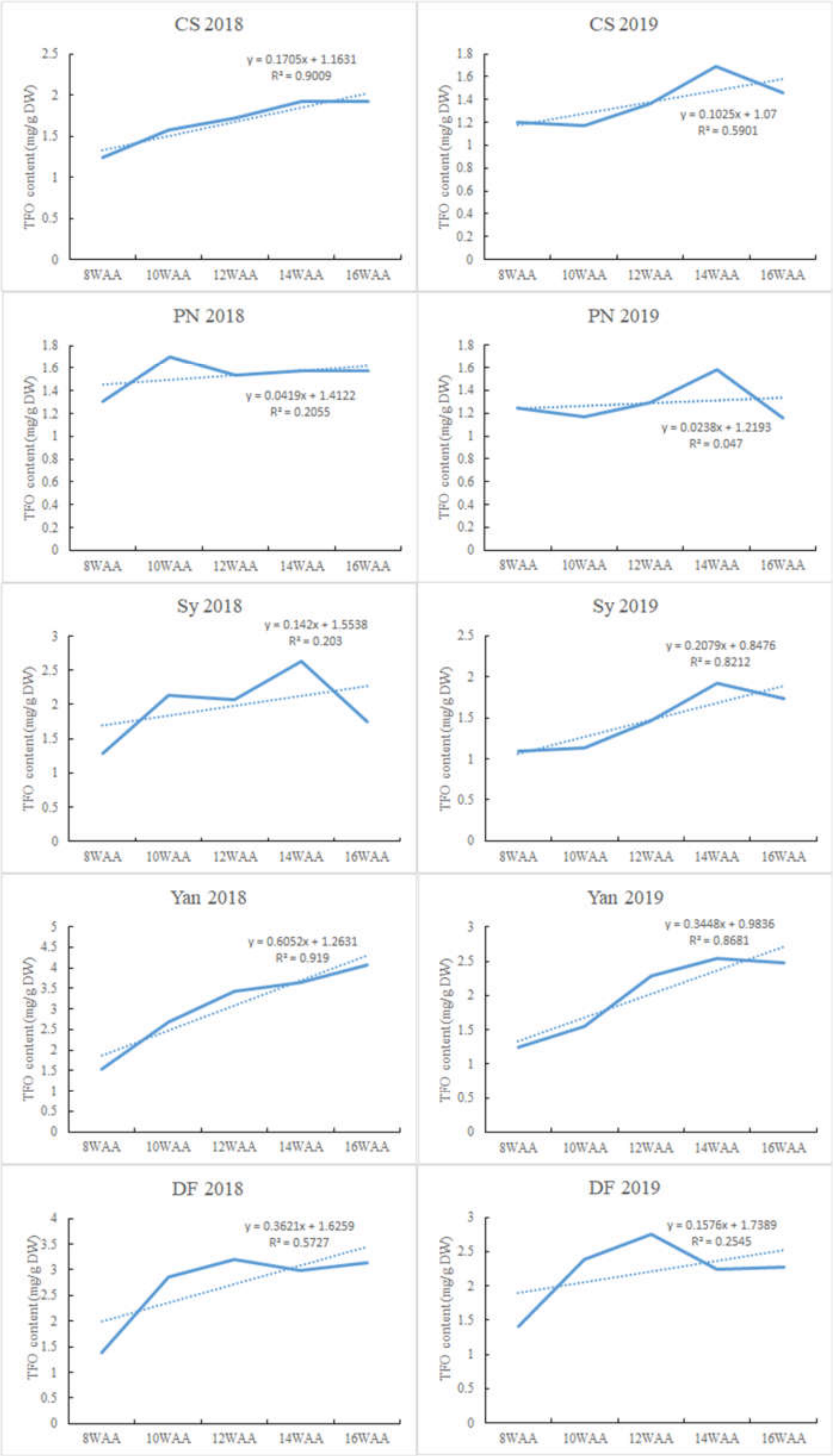

Figure S2 Regression analysis of TFO content.
